# GC–MS/MS Determination of Synthetic Cathinones: 4-chloromethcathinone, N-ethyl Pentedrone, and N-ethyl Hexedrone in Oral Fluid and Sweat of Consumers under Controlled Administration: Pilot Study

**DOI:** 10.3390/ijms24119387

**Published:** 2023-05-27

**Authors:** Melani Nuñez-Montero, Claudia Lombroni, Nunzia La Maida, Maria Concetta Rotolo, Simona Pichini, Esther Papaseit, Olga Hladun, Mireia Ventura, Lourdes Poyatos, Clara Pérez-Mañá, Magí Farré, Emilia Marchei

**Affiliations:** 1 Department of Clinical Pharmacology, Hospital Universitari Germans Trias i Pujol and Institut de Recerca Germans Trias i Pujol (HUGTiP-IGTP), 08916 Badalona, Spain; mnunezmon.germanstrias@gencat.cat (M.N.-M.); epapaseit.germanstrias@gencat.cat (E.P.); ohladun.germanstrias@gencat.cat (O.H.); lpoyatos@igtp.cat (L.P.); mfarre.germanstrias@gencat.cat (M.F.); 2 Department of Pharmacology, Therapeutics and Toxicology, Universitat Autònoma de Barcelona, 08193 Cerdanyola del Vallés, Spain; 3 National Centre on Addiction and Doping, Istituto Superiore di Sanità, 00161 Rome, Italy; claudia.lombroni@edu.unito.it (C.L.); nunzia.lamaida@iss.it (N.L.M.); mariaconcetta.rotolo@iss.it (M.C.R.); simona.pichini@iss.it (S.P.); emilia.marchei@iss.it (E.M.); 4 Department of Chemistry, Univesità degli Studi di Torino, Via Pietro Giuria 5, 10125 Torino, Italy; 5 Energy Control, Associació Benestar i Desenvolupament, 08012 Barcelona, Spain; mireia@energycontrol.org

**Keywords:** synthetic cathinones, gas-chromatography tandem mass spectrometry, oral fluid pharmacokinetics, sweat, 4-chloromethcathinone, N-ethyl pentedrone, N-ethyl hexedrone

## Abstract

This study presents a validated GC-MS/MS method for the detection and quantification of 4-chloromethcathinone or clephedrone (4-CMC), N-ethyl Pentedrone (NEP), and N-ethyl Hexedrone (NEH, also named HEXEN) in oral fluid and sweat and verifies its feasibility in determining human oral fluid concentrations and pharmacokinetics following the administration of 100 mg of 4-CMC orally and 30 mg of NEP and NEH intranasally. A total of 48 oral fluid and 12 sweat samples were collected from six consumers. After the addition of 5 μL of methylone-d_3_ and 200 μL of 0.5 M ammonium hydrogen carbonate, an L/L extraction was carried out using ethyl acetate. The samples, dried under a nitrogen flow, were then derivatized with pentafluoropropionic anhydride and dried again. One microliter of the sample reconstituted in 50 μL of ethyl acetate was injected into GC-MS/MS. The method was fully validated according to international guidelines. Our results showed how, in oral fluid, the two cathinones taken intranasally were absorbed very rapidly, within the first hour, when compared with the 4-CMC which reached its maximum concentration peak in the first three hours. We observed that these cathinones were excreted in sweat in an amount equivalent to approximately 0.3% of the administered dose for 4-CMC and NEP. The total NEH excreted in sweat 4 h after administration was approximately 0.2% of the administered dose. Our results provide, for the first time, preliminary information about the disposition of these synthetic cathinones in the consumers’ oral fluid and sweat after controlled administration.

## 1. Introduction

Synthetic cathinones are laboratory-synthetized stimulants that are chemically similar to cathinone, a substance found in the khat plant (*Catha edulis*) [1,2]; they can be much more potent than the natural product and, in some cases, very dangerous. [1,3]. Synthetic cathinones are sold as “legal” alternatives to controlled stimulants such as amphetamine and MDMA. Although the trends in synthetic cathinones reported to the EMCDDA are decreasing [4], cathinones dominated the seizures made in Europe in 2020 [5]; this group of new psychoactive substances is still posing a health threat [6].

Since their first appearance on the recreational drug market around 2010, synthetic cathinones have undergone numerous chemical modifications, primarily in an attempt to evade law enforcement. When the first generation of synthetic cathinones (methylone, mephedrone, and methylenedioxypyrovalerone [MDPV]) became illegal, a second generation (pentedrone, hexedrone, and their N-ethyl derivate) emerged [7].

Due to the novelty of these drugs, the data in the scientific literature on acute and chronic toxicity are still poorly understood or even completely unknown [6]. There are problems associated with the abuse of these substances; toxicity problems have been found, both at a cardiovascular level and at a neurological level, including the development of arrhythmia, hypertension, hyperthermia, agitation, confusion, psychosis, and coma [8,9]. In recent years, several cases of intoxication and death have been attributed to these substances [4,6,10,11,12,13]. Understanding the mechanisms of action of these drugs and the knowledge of their short- and long-term effects, their pharmacokinetic properties, and the correlation between the concentration in biological fluids and their activity has become crucial for public health reasons.

Oral fluid for short detection windows and sweat for medium detection windows are quickly becoming attractive alternative biological fluids for detecting the intake of drugs of abuse due to the relative ease of their noninvasive collection and for the detection of parent drugs. The amount of unbound drug in the bloodstream, the matrix pH, the substance pKa, and the lipophilicity all affect the excretion of substances into oral fluid and sweat [14].

While the determination of synthetic cathinones in oral fluid by gas chromatography (GC) or liquid chromatography (LC) coupled to mass spectrometry systems (GC-MS and LC-MS or LC-MS/MS) is reported in the literature [15,16,17,18,19,20,21,22,23,24,25,26], synthetic cathinones in sweat specimens remain scarcely explored [27]. Moreover, no determination of synthetic cathinones by GC-MS/MS is reported in the international literature.

The objective of the study presented here was to set up and develop a GC-MS/MS method for the simultaneous quantitation of the last generation of synthetic cathinones (4-chloromethcathinone, N-ethyl Pentedrone, and N-ethyl Hexedrone) in two non-conventional matrices: oral fluid and sweat. The developed method was then applied to investigate the concentrations and time courses in the consumers’ oral fluid after a single orally and intranasally self-administrated dose and to evaluate, for the first time, the excretion of these synthetic cathinones in sweat.

## 2. Results

### 2.1. Method Development and Validation

**Figure 1:**
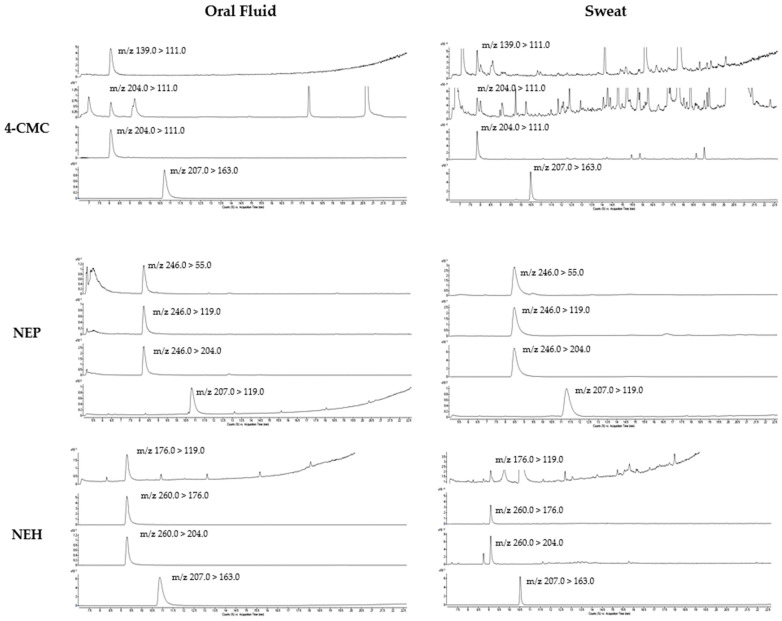
Chromatograms of an extract of 50 μL oral fluid spiked with 1.75 ng 4-CMC, NEP, and NEH and one sweat patch spiked with 10.0 ng of 4-CMC, NEP, and NEH.

For the compounds under investigation, which had an R^2^ value ≥0.990 and passed Mandel’s test, the linearity in both biological matrices was confirmed. The LOD and LOQ values were adequate for the purposes of the present study. The linearity, calibration results, LOD, and LOQ are shown in Table 1. The LOQ was set as the lowest calibrator point for each analyte and the representative chromatograms are shown in Figure 1.

The intra- and inter-assay accuracy and precision values were always lower than 20%; the recovery and the results are shown in Table 2.

**Figure 2:**
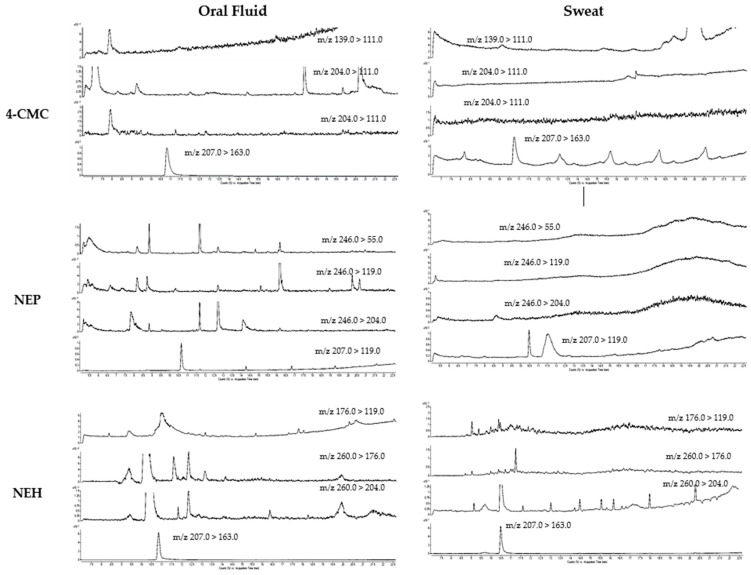
Extracted ion chromatograms of a drug-free oral fluid and sweat patch sample.

There were no interfering peaks in any of the analyzed oral fluid and sweat pool samples (Figure 2).

The dilution integrity was evaluated, and the over-curve sample concentrations gave values that were always within ±20% of the target for all the compounds. Regarding the freeze/thaw stability assays for the quality control samples, no significant degradation was observed after any of the three freeze/thaw cycles; the differences in concentration compared to the initial concentration were lower than 15%. Similar results (with differences always lower than 15%) were obtained in the case of the long-term stability tests, assuring the validity of the stored samples analysis. After one day (24 h) at room temperature (short-term stability test), 4-CMC showed degradation of greater than 30.0%, and NEP and NEH showed degradation of less than 15.0%.

### 2.2. Pharmacokinetics of 4-CMC, NEP, and NEH in Oral Fluid

**Figure 3:**
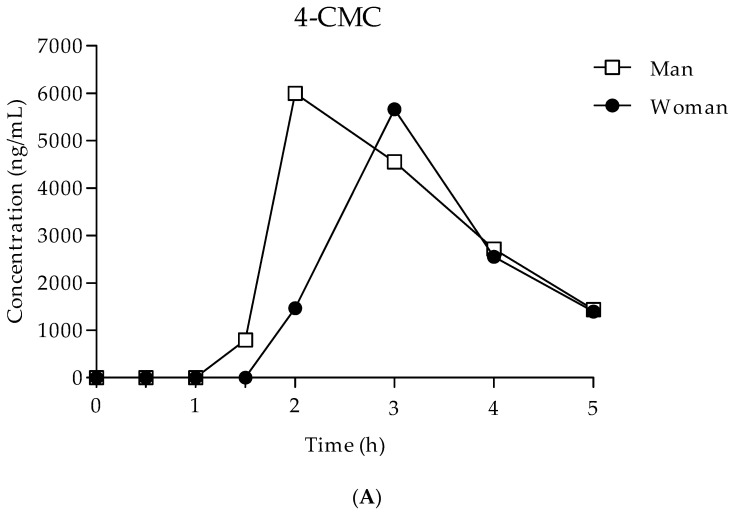

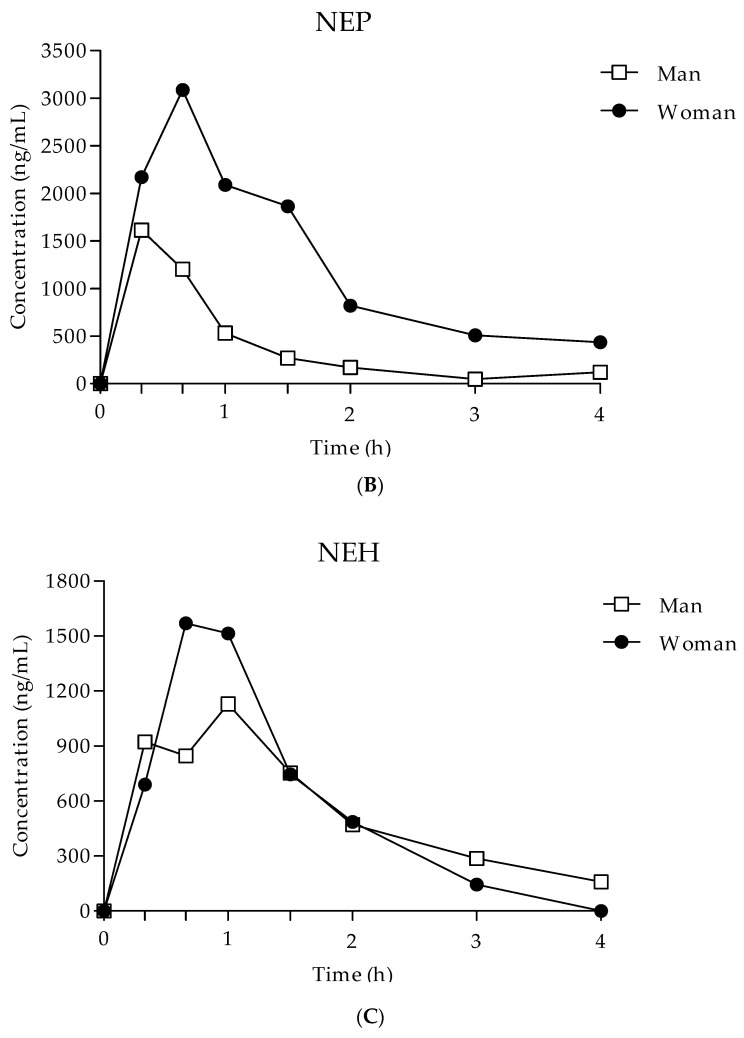
Time–concentration profiles for oral fluid (**A**) 4-CMC, (**B**) NEP, and (**C**) NEH.

**Figure 4:**
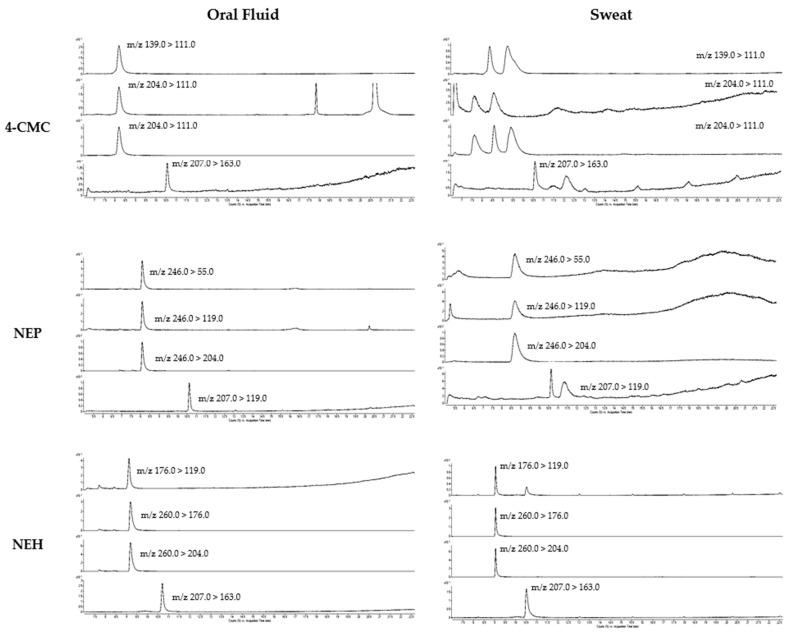
Extracted ion chromatograms of authentic samples of oral fluid and sweat patch.

The oral fluid concentration–time curves for the six consumers after the oral administration of 100 mg of 4-CMC and the intranasal administration of 30 mg of NEP or 30 mg of NEH are shown in Figure 2. The pharmacokinetic parameters derived from the data depicted in Figure 3 are presented in Table 3. The chromatograms of the real samples of oral fluid are shown in Figure 4.

In the oral fluid of the 4-CMC consumers, the maximum peak (Cmax), which was reached at an average time of around 2.5 h, appears similar, while the AUC_0–5_ in subject 2 was slightly higher (1.3-fold) than that of subject 1, despite being of the same dosage. After the absorption phase, the concentration of 4-CMC decreased 5 h after administration to concentrations of 1435.4 and 1398.5 ng/mL in subjects 1 and 2, respectively.

The concentration–time curves of NEP in the oral fluid of the consumer showed a fast peak concentration in subject 4 that was nearly double that observed in subject 3 (time peaks at 0.66 and 0.33 h, respectively). Then, the concentration decreased after 4 h, reaching the concentrations of 119.8 ng/mL and 437.0 ng/mL in subjects 3 and 4, respectively. Despite the same dose (30 mg) and the same route of administration (intranasal), the AUC_0–4_ in subject 4 was 3.2-fold higher than that in subject 3.

The time peaks of NEH occurred at 0.66 and 1 h from the start of intranasal administration with Cmax and were similar in both subjects (Table 3); then, the peak decreased to undetectable values within the next 4 h in subject 6 and to 159.7 ng/mL in subject 5. At the same dose (30 mg) and route of administration (intranasal), the two subjects had similar AUC_0–4_.

After reaching peak exposure, the oral fluid disposition of the synthetic cathinones under investigation had a salivary T_1/2_ of 1.2 h for nearly all the subjects enrolled in the study. Subjects 3 and 6 were the exceptions, with a T_1/2_ of 0.9 h and a T_1/2_ of 0.6 h, respectively.

### 2.3. 4-CMC, NEP, and NEH in Sweat

Table 4 shows the sweat concentration of the synthetic cathinones under investigation over a 4 or 5 h period from the subjects administered 100 mg of 4-CMC orally and 30 mg of NEP or 30 mg of NEH intranasally. The chromatograms of the real sweat samples are shown in Figure 4.

After administration, 4-CMC was detected in only one of the two subjects; this was also the case after NEH administration. NEP was detected 4 h post administration in both the subjects enrolled in this study.

The total amount of drug excreted in the first 4 or 5 h was estimated using the concentrations of the synthetic cathinones under investigation in the patches 4 or 5 h post administration and the ratio between the area of the patch and total body surface area of each subject.

The 4-CMC excreted in sweat after the administration was 0.3 mg, which was equivalent to about 0.3% of the orally administered dose. The mean total NEP excreted in sweat after the administration was 0.09 mg (subject 3: 0.07 mg; subject 4: 0.11 mg), which was equivalent to about 0.3% of the intranasally administered dose. The total NEH excreted in sweat 4 h after the administration was 0.06 mg, which was equivalent to about 0.2% of the intranasally administered dose.

## 3. Discussion

The GC-MS/MS method developed in the present study for the determination of NEH, NEP, and 4-CMC highlighted the presence of cathinones in the oral fluid and, for the first time, in the sweat of consumers and was validated in terms of accuracy, precision, and robustness in accordance with the internationally required parameters [28,29].

In general, intranasal intake, smoking, and sublingual intake produce a rapid absorption of the substance and therefore a rapid effect. After oral administration, on the other hand, absorption can be highly variable because it is influenced by various factors such as the overcoming of the intestinal barrier, gastric acidity, the simultaneous intake of food and gastric emptying, or the presence of other drugs. Our results showed how the two cathinones taken intranasally were absorbed very rapidly, within the first hour, when compared with the 4-CMC, which reached its maximum concentration peak in the first three hours. The 4-CMC data were in agreement with that which was reported for similar orally administered substances, such as mephedrone (Tmax 2.3 h) and methylone (Tmax 2 h) [17,18,30].

After reaching peak exposure, the oral fluid dispositions of 4-CMC and NEP had a similar mean T_1/2_ of 1.2 h and 1.1 h, respectively, while that of NEH had a mean T_1/2_ of 0.9 h.

The comparison with AUC_0–4_ shows how, with the same administered dose and route of administration, the amount of NEP was about 1.5 times higher than that of NEH. A possible gender difference was observed for NEP in Tmax and AUC, with much higher concentrations in woman; inter-subject variability could explain the difference.

Even if few real samples (only three males and three females, which was insufficient to explore sex differences) were made available to prove the robustness of the analytical methods, these first preliminary data show different pharmacokinetics, particularly for NEP and NEH, despite the same administered dose and the same route of administration. However, these results must be confirmed in more subjects and doses in future studies, mainly to understand the type of kinetics: linear or not. An another limitation of the present study is that no metabolites were measured due to the unavailability of pure chemical standards at the time of the study. Although this study included participants of both sexes, the small number of females was insufficient to explore the sex differences related to the acute effects of methylone.

Regardless of which route of administration was used, all the synthetic cathinones under investigation were found in sweat, with the exception of that of subject 2 and subject 5. Different perspiration rates, volumes of sweat, and effects of drug pharmacological activity on body temperature are likely to explain the interindividual variations in excretion of the same dose of consumed cathinones. Unfortunately, in our study sweat patch collection was carried out up to 5 h after administration. This is a study limitation since, as reported in our previous studies on amphetamine-like substances [27,31,32], where collection lasted up to 24 h, the maximum excretion in sweat for these compounds was indeed observed 24 h after administration.

The total synthetic cathinones excreted in sweat after the administration of different drug doses and routes of administration ranged between 0.3 mg for 4-CMC, 0.09 mg for NEP, and 0.06 mg for NEH, which was equivalent to about 0.3% of the administered doses for 4-CMC and NEP and 0.2% of the administered doses for NEH. Although this is a preliminary study involving few subjects, the obtained results are clear evidence that even with a single administration of synthetic cathinones, the parent drugs can be detected in sweat, which can be of use for clinical and forensic purposes. The use of sweat patches provides an alternative matrix with which to assess drug use in consumers without invasive sample collection. The sweat patch analysis provided the physicians with information about recent drug consumption, which can be used for patient-specific therapy.

## 4. Materials and Methods

### 4.1. Chemicals and Materials

4-chloromethcathinone (Clephedrone, 4-CMC), N-ethyl Pentedrone (α-Ethylaminopentiophenone, NEP), N-ethyl Hexedrone (α-Ethylaminohexanophenone, NEH), and Methylone-d_3_ (deuterated internal standard, IS) were purchased from Cayman (Ann Arbor, MI, USA). Pentafluoropropionic anhydride (PFPA) was supplied from Merk Life Science SRL (Milano, Italy).

LC-MS/MS grade water, methanol, acetonitrile, LC-MS grade formic acid and ethyl acetate, ammonium hydrogen carbonate and sodium hydroxide were purchased from Carlo Erba (Cornaredo, Italy).

### 4.2. Calibrators and Quality Control (QC) Solutions

Stock standard solutions (1 mg/mL) were prepared in methanol. Working solutions at concentrations of 100, 10, and 1 µg/mL were prepared by dilution of the stock standards with methanol and stored at −20 °C until analysis. The internal standard (IS) working solution was used at a concentration of 10 µg/mL.

Drug-free oral fluid was collected from 10 healthy donors, analyzed during the method validation to eliminate sources of chromatographic interference, and mixed to obtain a homogeneous pool of blank samples to be used for calibration standards and quality control (QC) samples. Drug-free sweat was collected by applying a sweat patch to the back of a healthy donor, cleaning the skin with 70% isopropyl alcohol, and removing it 5 h after application. All biological samples were frozen at −20 °C until analysis. No preservatives were added to the specimens.

Calibration standards containing 35, 50, 100, 500, 1000, 3000, and 5000 ng/mL oral fluid or 10, 25, 50, 100, 250, and 500 ng/patch were prepared daily for each analytical batch by adding suitable amounts of methanol working solutions to 50 µL of pre-checked drug-free oral fluid or sweat patches. Quality control (QC) samples of 4500 ng/mL or 450 ng/patch (high control), 1500 ng/mL or 250 ng/patch (medium control), 45 ng/mL, or 15 ng/patch (low control), and samples at the LOQ of each analyte were prepared in drug-free oral fluid, aliquoted, and stored at −20 °C.

### 4.3. Participants

The oral fluid and sweat used in this study were obtained from six consumers, 3 females (24, 25, and 36 years old with BMIs of 19.3, 19.7, and 27.5, respectively) and 3 males (26, 28, and 32 years old with BMIs of 19.2, 21.6, and 24.8, respectively). All the participants were Caucasians and had had recreational experience with psychostimulants, such as cocaine, amphetamines, MDMA, and other synthetic cathinones (orally and intranasally). All were able to snort the substances. The participants were recruited by word of mouth and snowball sampling through the harm reduction, non-governmental organization Energy Control (ABD).

The local human research ethics committee (CEI-HUGTiP ref. PI-18-267) to investigate the potential for abuse and the human pharmacology of substances of abuse, including synthetic cathinones and cannabinoids, approved the study. It was conducted according to the Declaration of Helsinki recommendations and Spanish law on clinical investigation. All the participants were informed, both orally and in writing, and signed informed consent prior to inclusion. The participants received monetary compensation for their participation.

The study design was naturalistic, prospective, and observational, with minimal intervention. The sessions took place on three different days, one for each substance, at a private club with ambient music. The ambient temperature in the private club was around 24 °C. The sessions started at 3:00 p.m. and finished at 8:00–9:00 p.m. All the doses were self-administered and were also self-selected by each participant, based presumably on their previous experience. The doses were in the range of those recommended in risk reduction organizations. The drug samples were tested by Energy Control, a harm reduction organization that provides a drug checking service for users. Measures of the pharmacological effects were collected (data not presented in this manuscript). The study methodology was similar to that of other previously published studies [17].

### 4.4. Design, Treatments and Collection

The six consumers were divided into three study groups (group 1: 4-CMC; group 2: NEP; and group 3: NEH). The subjects of each group (*n* = 2) self-administered a cathinone: group 1 orally (100 mg of 4-CMC) and groups 2 and 3 intranasally (30 mg of NEP or NEH, respectively).

Prior to the study session, the participants underwent a general medical examination and a psychiatric interview. They did not refer to a previous or actual history of diseases or mental disorders. The general medical examination and psychiatric interview did not show actual or previous medical disorders or psychiatric disorders, including substance use disorder (DSM-V). The participants were requested to abstain from consumption of any drug of abuse during the week before the study. The abstinence was verified by the performance of urine drug testing before administration (for benzodiazepines, MDMA, morphine, tetrahydrocannabinol, methadone, amphetamine, methamphetamine, cocaine, tricyclic antidepressants, and barbiturates, using the Drug-Screen Multi 10TD Test [Multi-Line] (Nal Von Minden, Moers, Germany). All urine samples were negative at the baseline.

Samples of oral fluid were collected at the baseline (pre-dose) and at 0.5, 1, 1.5, 2, 3, 4, and 5 h after oral self-administration of 4-CMC and at 0.33, 0.66, 1, 1.5, 2, 3, and 4 h after intranasal self-administration of NEP or NEH. Approximately 0.5 mL of oral fluid was typically collected with standard Salivette^R^ tubes, and the samples were immediately stored at −20 °C until analysis.

The patches for sweat collection were placed on the backs of the subjects, under the scapula, 4 h before administering the substance (−4–0H) and up to 5 h after oral administration (0–5H) of 4-CMC and 4 h (0–4H) after intranasal administration of NEP or NEH. After removal, the patches were labelled and stored in plastic bags at −20 °C until analysis.

All the selected doses were well tolerated, and no serious adverse events were observed. No local tissue damage to the nostrils or any other potential acute medical complication after snorting was reported.

### 4.5. Sample Preparation

The extraction procedure was tested with fortified oral fluid or a sweat patch using different extraction solvents (ethyl acetate, chloroform, and a 9:1, *v*/*v* chloroform/isopropanol mixture) and different derivatization reagents (Bis(trimethylsilyl) trifluorocetamide, N-Methyl-bis(trifluoroacetamide, and pentafluoropropionic anhydride). Ethyl acetate and PFPA for the extraction and derivatization of synthetic cathinones under investigation were found to be the best compromise for our analytical purposes.

Fifty microliters of oral fluid was spiked with 5 μL of IS solution (methylone-d_3_ at concentration of 10 μg/mL). Next, 200 μL of 0.5 M ammonium hydrogen carbonate and one drop of 0.1 M sodium hydroxide were added. Liquid–liquid extraction (LLE) was performed with 2 mL of ethyl acetate by horizontal shaker mixing for 5 min. After centrifugation at 4000 rpm for 5 min, the organic layer was transferred to another tube and the solvent was evaporated to dryness under a stream of nitrogen.

The residue obtained was then derivatized with 25 μL of PFPA and 25 μL of ethyl acetate for 15 min at 70 °C. The mixture was dried again under nitrogen and reconstituted in 50 μL of ethyl acetate. A 1 μL quantity of the derivatized sample was then injected into the GC-MS/MS system.

For the cathinone determination in sweat, the absorbent pad removed from the patch with clean tweezers was added to 5 μL of IS working solution in a clean tube and was extracted, after a vortex mixing with 1 mL 0.5 M ammonium hydrogen carbonate, with 2 mL of ethyl acetate by horizontal shaker mixing for 5 min. After centrifugation at 4000 rpm for 5 min, the organic layer was transferred to another tube and the solvent was evaporated to dryness under a stream of nitrogen. The dried sample was derivatized as described above. A 1 μL quantity of the derivatized sample was then injected into the GC-MS/MS system.

### 4.6. GC-MS/MS Conditions

Analysis was performed on a 7890B GC system equipped with a multimode inlet (MMI) and a 7693A Automatic Sampler (all from Agilent Technologies, Santa Clara, CA, USA). The capillary column used was an Agilent Technologies HP-5MS UI (30 m × 0.25 mm × 0.25 μm). The samples were injected in pulsed splitless mode with the injector port temperature of 270 °C. Helium was used as a quenching gas at a flow of 2.25 mL/min The column temperature was initially set at 80 °C for 1 min before increasing to 160 °C at 30 °C/min and then increasing again to 250 °C at 5 °C/min (held for 1 min), taking the total run time to 22.67 min. The GC instrument was interfaced to a GC/MS 7000D triple quadrupole mass spectrometer (Agilent Technologies, Santa Clara, CA, USA). The MS analyses were conducted in positive electron ionization (EI) mode. The transfer line and ion source temperature were set at 280 °C. Nitrogen was used as the collision-induced dissociation (CID) gas for ion fragmentation at a flow of 1.5 mL/min. Quantifications were performed using multiple reaction monitoring (MRM) transitions. Two transitions for each analyte and one transition for the deuterated standards were selected. The MRM transitions and collision energies of the corresponding quantifier and qualifier ions for each compound and IS are listed in Table 5.

### 4.7. Method Validation

Prior to application to real samples, the method was tested using a four-day validation protocol following the most recent criteria for bioanalytical method development and validation [28,29,33]. Analytical bias, imprecision, limit of detection (LOD), limit of quantification (LLOQ), linearity, carryover, and recovery (RE) were assessed.

Repeated measurements of the oral fluid or sweat patch calibration point concentrations were used in order to perform a linearity experiment. To create calibration curves, peak area ratios of the analyte and internal standard were plotted against the analyte concentrations. Moreover, Mandel’s test (Fcalc) was performed. The coefficient of determination (R^2^) ≥ 0.990 and Fcalc values lower than tabulated limit (Ftab, 95%) were considered evidence of good linearity. For the over-curve samples with amounts that were 5 and 10 times higher than the highest calibrators, the dilution integrity was examined.

Ten individual drug-free oral fluid or sweat patch samples were extracted and the standard deviation (σ) of the blank responses was evaluated for the determination of the limit of detection (LOD) (the smallest amount or concentration of the analyte that can be reliably distinguished from zero) and the limit of quantification (LOQ) (the lowest concentration of the analyte that can be measured with an acceptable repeatability and trueness). A minimum requirement for signal-to-noise ratios of 3 and 10 is widely accepted for LOD and LOQ, respectively.

Absolute analytical recoveries were tested by comparing the peak areas obtained when the quality control samples (five drug-free oral fluid or sweat patch samples for all three QC levels) were analyzed by adding the working solutions to the extracts of drug-free oral fluid or sweat patch samples prior to and after the extraction procedure.

The potential for carryover was investigated by injecting extracted drug-free oral fluid or sweat patch samples after analysis of the highest concentration point of the calibration curve and the measurement of the area of eventual peaks, which were present at the retention times of the analytes under investigation.

A total of five replicates at each of the QC concentrations added to the drug-free oral fluid or sweat patches, extracted as reported above, were analyzed on the same day for the determination of intra-assay precision and accuracy. The inter-assay precision and accuracy were determined for three independent experimental assays of the aforementioned replicates. Precision and accuracy, expressed as relative standard deviation (RSD or CV) and relative error of the measured concentrations (%), respectively, were expected to be within ±20%.

The effect of three freeze/thaw cycles (the QC samples were stored at −20 °C for 1, 2, and 4 h) on the stability of the compounds in the oral fluid and sweat patches was evaluated by repeated analysis (*n* = 3) of the QC. A short-term stability test was performed by keeping the QC samples at room temperature for one day (24 h). A long-term stability test was performed by re-analyzing the replicates of three real oral fluid samples and three sweat patches once a month for a 3-month period. The stability was expressed as a percentage of the initial concentration (first analyzed batch, 0 h) of the analytes in both the QC and the real samples.

### 4.8. Pharmacokinetic Parameters

With regard to the oral fluid of the synthetic cathinones under investigation, the following parameters were determined after drug administrations: peak concentration (Cmax_0–4_ or Cmax_0–5_) and time taken to reach peak concentration (Tmax_0–4_ or Tmax_0–5_) were directly obtained from the concentration–time curves. The area under the oral fluid concentration–time curve (AUC_0–4_ or AUC_0–5_) was calculated by the linear trapezoidal rule.

The terminal-phase elimination half-life (T_1/2_) was calculated as 0.693/Ke, where Ke (elimination rate constant) was the slope of the apparent elimination phase of the natural logarithmic (ln) transformation of the oral fluid concentration–time curve, which was estimated using linear regression.

## 5. Conclusions

We developed and validated the analytical method for quantifying 4-chloromethcathinone (clephedrone), N-ethyl Pentedrone, and N-ethyl Hexedrone in human oral fluid and, for the first time, in sweat. The extraction and analysis method for the detection of all the analytes under investigation was simple and was fully validated.

This study added to the literature on the preliminary information about synthetic cathinone clinical pharmacokinetics and provided the first data on their excretion in sweat. These matrices are an alternative for both clinical and toxicological requests when blood or urine are unavailable, allowing for the detection of a recent drug assumption and the gathering of information in a simple, speedy, and non-invasive manner.

Considering the obtained results, further studies are needed to better understand the pharmacokinetics of synthetic cathinones and the potential impact of these new drugs on human health. Furthermore, future studies will evaluate the pharmacological effects of these synthetic cathinones in humans to assess their abuse potential.

## Figures and Tables

**Table 1:** Linearity values, lower limit of detection (LOD) lower limit of quantification (LOQ), and Mandel’s fitting test (Fcrit95%) for analytes under investigation in oral fluid and sweat samples.

Compound	Linear Range ^a^	Linear Equation	R^2^	LOD	LOQ	*p*-Value	Fcalc	Fcrit95%
**Oral fluid**								
4-CMC	35–5000	y = 0.0018x + 0.0829	0.991 ± 0.001	12	35	0.888	3.940	4.242
NEP	35–5000	y = 0.0015x − 0.0189	0.990 ± 0.002	12	35	0.944	0.820	4.242
NEH	35–5000	y = 2.698x + 0.1444	0.993 ± 0.004	12	35	0.775	3.490	4.242
**Sweat**								
4-CMC	10–500	y = 0.0617x − 0.5193	0.997 ± 0.002	3	10	0.135	3.660	4.543
NEP	10–500	y = 0.0648x − 0.6198	0.993 ± 0.006	3	10	0.202	2.650	4.543
NEH	10–500	y = 0.2609x − 1.3557	0.997 ± 0.001	3	10	0.080	2.790	4.543

^a^ Concentration was expressed in ng/mL for oral fluid or ng/patch for sweat.

**Table 2:** Accuracy, precision, and recovery values for analytes under investigation in oral fluid and sweat samples.

Compound	Intra-Assay Accuracy (%)			Inter-Assay Accuracy (%)			Intra-Assay Precision (%CV)			Inter-Assay Precision (%CV)			Recovery (%)		
QC	L	M	H	L	M	H	L	M	H	L	M	H	L	M	H
**Oral fluid**															
4-CMC	7.5	4.1	13.9	7.7	15.3	6.1	1.6	6.0	6.9	3.9	6.7	7.3	82.5 ± 1.5	84.2 ± 2.1	86.9 ± 4.7
NEP	11.6	5.9	12.0	15.2	13.9	10.2	14.8	8.5	1.7	13.3	9.2	1.7	85.6 ± 3.7	92.7 ± 4.6	90.4 ± 1.0
NEH	15.5	11.0	8.3	10.6	11.5	11.0	0.5	15.4	4.5	2.7	15.4	4.8	90.9 ± 3.3	94.9 ± 6.8	95.5 ± 1.3
**Sweat**															
4-CMC	16.1	6.7	9.7	13.4	7.02	14.7	1.5	8.7	6.6	3.2	9.3	9.7	98.8 ± 1.1	95.3 ± 2.5	99.4 ± 2.5
NEP	8.9	13.0	7.1	8.5	16.6	6.7	5.7	13.4	1.8	8.6	8.5	2.9	90.5 ± 1.7	89.2 ± 3.3	97.6 ± 2.4
NEH	17.3	7.6	9.1	15.3	6.3	4.4	0.4	13.6	11.2	6.6	11.5	9.1	88.1 ± 2.3	97.4 ± 3.1	98.3 ± 0.5

Abbreviations: QC = quality control; L = low quality control concentration (45 ng/mL oral fluid, 15 ng/patch); M = medium control concentration (1500 ng/mL oral fluid, 250 ng/patch); H = high control concentration (4500 ng/mL oral fluid, 450 ng/patch).

**Table 3:** Pharmacokinetic parameters of 4-CMC, NEP, and NEH in oral fluid.

Substances		Subject 1 (Man)	Subject 2 (Woman)
4-CMC (oral, 100 mg)	Cmax (ng/mL) Tmax (h) AUC_0–5_ (h·ng/mL) T_1/2_ (h)	6002.3 2.0 12,886.6 1.2	5663.1 3.0 10,014.0 1.2
		**Subject 3 (man)**	**Subject 4 (woman)**
NEP (intranasal, 30 mg)	Cmax (ng/mL) Tmax (h) AUC_0–4_ (h·ng/mL) T_1/2_ (h)	1613.1 0.33 1529.3 0.9	3086.3 0.66 4903.4 1.2
		**Subject 5 (man)**	**Subject 6 (woman)**
NEH (intranasal, 30 mg)	Cmax (ng/mL) Tmax (h) AUC_0–4_ (h·ng/mL) T_1/2_ (h)	1130.7 1.0 2160.7 1.2	1569.1 0.66 2272.5 0.6

Abbreviations: Cmax: peak concentration; Tmax: time taken to reach peak concentration; AUC: area under curve; T_1/2_: elimination half-life time.

**Table 4:** Concentrations in sweat samples from subjects following oral administration of 100 mg (subjects 1 and 2) of 4-CMC and intranasal administration of 30 mg (subjects 3 to 6) of NEP or NEH, respectively.

Substances	Time (h)	Subject 1 (Man)	Subject 2 (Woman)
4-CMC (oral, 100 mg)	5 h post administration	153.7 ng/patch	ND
		**Subject 3 (man)**	**Subject 4 (woman)**
NEP (intranasal, 30 mg)	4 h post administration	40.1 ng/patch	71.7 ng/patch
		**Subject 5 (man)**	**Subject 6 (woman)**
NEH (intranasal, 30 mg)	4 h post administration	ND	34.4 ng/patch

Abbreviations: ND, not determined.

**Table 5:** Retention times (RT) and mass spectrometry parameters for all target compounds.

Compound	RT	MRM Transition			
		Quantifier (*m*/*z*)	CE ^a^ (eV)	Qualifier (*m*/*z*)	CE ^a^ (eV)
4-CMC	8.1	204 > 160	10	204 > 119 138 > 111	25 15
NEP	8.3	246 > 119	30	246 > 55 246 > 204	30 10
NEH	9.3	260 > 204	10	260 > 176 176 > 119	25 10
Methylone-d3 (IS)	11.0	207 > 163 ^b^ 207 > 119 ^c^	10	-	-

^a^ CE = collision energy; ^b^ MRM used for 4-CMC and NEH; ^c^ MRM used for NEP determination.

## Data Availability

All data generated or analyzed during this study are included in this published article.
